# EBV Reactivation and Chromosomal Polysomies: *Euphorbia tirucalli* as a Possible Cofactor in Endemic Burkitt Lymphoma

**DOI:** 10.1155/2012/149780

**Published:** 2012-04-24

**Authors:** Susanna Mannucci, Anna Luzzi, Alessandro Carugi, Alessandro Gozzetti, Stefano Lazzi, Valeria Malagnino, Monique Simmonds, Maria Grazia Cusi, Lorenzo Leoncini, Cornelia A. van den Bosch, Giulia De Falco

**Affiliations:** ^1^Department Human Pathology and Oncology, University of Siena, 53100 Siena, Italy; ^2^Department of Oncology, Hematology, University of Siena, 53100 Siena, Italy; ^3^Pilgrims' Hospice, Margate, Kent CT94AD, UK; ^4^Department Biotechnology, Section of Microbiology, University of Siena, 53100 Siena, Italy

## Abstract

Burkitt lymphoma is endemic in the Equatorial Belt of Africa, its molecular hallmark is an activated, *MYC* gene mostly due to a chromosomal translocation. Especially in its endemic clinical variant, Burkitt lymphoma is associated with the oncogenic Epstein-Barr virus (EBV), and holoendemic malaria acts as an amplifier. Environmental factors may also cooperate in Burkitt lymphomagenesis in the endemic regions, such as plants used as traditional herbal remedies. *Euphorbia tirucalli*, a plant known to possess EBV-activating substances, has a similar geographical distribution to endemic Burkitt's Lymphoma and is used as a hedge, herbal remedy and toy in the Lymphoma BeltI. In this study we aimed at determining if exposure to *Euphorbia tirucalli* could contribute to lymphomagenesis, and at which extent. Lymphoblastoid and cord blood-derived cell lines were treated with plant extracts, and the expression of EBV-coded proteins was checked, to assess EBV reactivation. The occurrence of chromosomal translocations was then investigated by FISH. Our preliminary results suggest that *E. tirucalli* is able to reactivate EBV and determine chromosomal alterations, which leads to c-MYC altered expression. The existence of genomic alterations might determine the accumulation of further genetic alteration, which could eventually lead to a transformed phenotype.

## 1. Introduction

Burkitt's Lymphoma (BL), a high-grade Non-Hodgkin's lymphoma, is endemic in the Lymphoma Belt of Africa, which lies between 10°N and 10°S of the Equator [1, 2]. Within these geographical boundaries, BL accounts for up to 70% of children cancer with rates up to 10 cases of Burkitt's Lymphoma per 100,000 children under the age of 14 years [3]. Burkitt's Lymphoma characteristically has a translocation involving a deregulated, activated, *MYC* gene on chromosome 8 and immunoglobulin genes on chromosome 14, or, more rarely, chromosomes 2 or 22 [1], though alternative pathogenetic mechanisms leading to *MYC* activation have also been described [4, 5]. Burkitt's Lymphoma is associated with the oncogenic Epstein-Barr virus [6], in particular, 98% of Burkitt's Lymphoma cases in the Lymphoma belt show positivity to EBV [7]. EBV is recognised as a Class 1 human carcinogen and is thought to play a pivotal role in lymphomagenesis in endemic Burkitt's Lymphoma [8]. Holoendemic malaria acts as an amplifier and has been shown to be able to activate the latent EBV in B-lymphocytes in children in the Equatorial Belt [9, 10]. The combination of malaria and early infection with the Epstein-Barr virus is thought to be responsible for boosting the incidence of Burkitt's lymphoma a hundred-fold in Africa, compared with rates in the France, and the USA [11, 12]. Children who subsequently develop the endemic Burkitt's Lymphoma have raised antibody levels to the EBV Viral Capsid Antigen (VCA) of EBV several years before they actually develop the tumour [13]. Raised levels of this antibody are also found in the relatives of children with Burkitt's Lymphoma [14] and in those who have used traditional herbal remedies [15], which have been shown to be capable of activating the EBV [16].

*Euphorbia tirucalli*, a plant known to possess EBV-activating substances, has a similar geographical distribution to endemic Burkitt's Lymphoma [17] and is used as a hedge, herbal remedy, and toy in the Lymphoma Belt. This plant is found significantly more often at the homes of Burkitt's Lymphoma patients [17, 18] and the incidence of Burkitt's Lymphoma has fallen in Northern Zambia following the eradication of thickets of *E. tirucalli *[19].

*Euphorbia tirucalli* possesses a milky, rubbery sap which contains a 4-deoxyphorbol ester [20] closely related to the tumour-promoter substance TPA (12-O-tetra-decanoy-phorbol-13-acetate), which is derived from another Euphorbia, *Croton tiglium*. These TPA-related extracts of *Euphorbia tirucalli* present in the plant are secreted into the soil around the plant in active form [21] and can activate the latent EBV within the cell [16, 22], enhance EBV-mediated cell transformation [21], and modulate EBV-specific T-cell activity [21], myelopoiesis, and cellular immunity [23]. EBV and TPA can work synergistically in nude mice to produce both T- and B-cell lymphomas [24]. TPA's activity is also potentiated by the association with nucleic acids. Other plants commonly found in the Lymphoma belt and elsewhere in the tropics, typically those belonging to the Euphorbiaceae, and Thymelaeaceae families, are also known to induce the EBV lytic phase [16]. Some, but not all of these Euphorbiae, contain identical or similar phorbol esters to those found in *E. tirucalli* [25]. EBV-activation has been shown to be dependent on cellular protein kinase C (PKC), irrespective of the extracts' tumour-promotion abilities [26]. The TPA-related substances present in *E. tirucalli* could be expected to exhibit similar properties to those of TPA.

A research paper of the early nineties reported that *E. tirucalli* extracts can induce chromosomal abnormalities when added to EBV-infected cord blood B-lymphocytes [27]. Aya et al. showed that the cells multiplied rapidly following exposure to the extracts and, after one year of culture, ten percent of the chromosomal abnormalities induced by these plant extracts affected chromosome 8 with activation of the oncogene *MYC*, thus reproducing the crucial translocation characterizing Burkitt's Lymphoma [27], and that cells treated with the Euphorbia extracts produced lymphomas when injected into nude mice [27].

 These observations suggest that *Euphorbia tirucalli* extracts and, possibly, extracts of other plants, which are known to have similar EBV-activating properties, in conjunction with other environmental factors, could play an important role in lymphomagenesis in endemic African Burkitt's Lymphoma. Despite the interesting observation reported by Aya in the early nineties, no further study since then has elucidated the molecular mechanisms by which some plant extracts may act as a cofactor in lymphomagenesis. Therefore, it is also interesting trying to explain the close link between the geographic distribution of *E. tirucalli* and the incidence of BL in the endemic areas. The aim of our study was to determine if exposure to *E. tirucalli* extracts could result in EBV reactivation and induction of genomic alteration, which might contribute to transformation. Therefore, we treated both a lymphoblastoid cell line (LCL) and a cord-blood- (CB-) derived cell line newly infected with EBV with this plant extracts. It was considered that the LCLs are likely to have accumulated preexisting genetic abnormalities [28] having been cultured for a number of years, whereas the cord-blood cell line should be a better *in vitro* model to mimic the effects of the plant extracts *in vivo* on EBV-infected cells. Cells were treated with different concentrations of *E. tirucalli*, and we monitored its effect on cell proliferation, EBV antigen expression, and induction of genomic alterations, such as chromosomal translocations.

 Our results indicate that exposure to the plant extracts is able to reactivate EBV from its latent phase, as indicated by the expression of the EBV Zebra antigen following the treatment. In addition, the expression of EBV early antigens was also observed, along with a marked up regulation of LMP1, EBNA1, and EBNA2. In addition, though the specific chromosomal translocations present in Burkitt's lymphoma were not detected after *E. tirucalli* exposure, we observed the occurrence of polysomies involving chromosome 8, as demonstrated by the existence of multiple signals for *MYC* by FISH, which may result in overexpression of c-MYC, both at the mRNA and the protein level. In addition, increased expression for BCL2 was also observed, even in the absence of any genetic translocations involving this gene.

 All together, our results suggest that *E. tirucalli*, through EBV reactivation and induction of genetic alterations leading to *MYC* overexpression, could contribute to the malignant transformation process.

## 2. Materials and Methods

### 2.1. Cell Lines and Cell Culture

The human lymphoblastoid cell line (LCL) was a kind gift of Prof. A. Lanzavecchia (IRB, Bellinzona, Switzerland). The human cord-blood- (CB-) derived cell line was obtained from cells newly infected and immortalized by EBV, following the protocol described by Pelloquin et al. [29]. Mononucleated cells were isolated from cord blood by Ficoll fractionation. After isolation, purified EBV obtained from B95.8 cell line (kindly provided by dr. M. Kleines, Austria) was added to the cell culture, in a 1 : 1 ratio. Cells were cultured in the presence of Cyclosporin A at a final concentration 2 *μ*g/mL. The efficiency of infection was demonstrated by cluster formation in the cell culture after an overnight incubation with the virus. The establishment of the cell line was achieved after one month. Immunophenotype of the established CB cell line (CD79^+^, CD34^−^, CD138^−^, IRF4^−^, BCL2^+^, and BCL6^−^) was assessed as described below. Before treatment, cell caryotype was also assessed to confirm the absence of chromosomal translocations and aneuploidies. An EBV-negative Burkitt lymphoma-derived cell line (Ramos) was also used and treated as follows. For daily experiments and treatment with *E. tirucalli*, cells were cultured in RPMI supplemented with 10% FBS, 1% l-glutamine, penicillin/streptomycin, with 5% CO_2_, at 37°C.

### 2.2. Immunocytochemisty

 Immunocytochemical studies (ICC) were performed on representative cell smears of both treated and untreated cells using microwave pretreatment or proteolytic digestion of slides for antigen retrieval. A large panel of antibodies (Table 1) recognizing the various EBV antigens was applied, in conjunction with the streptavidine-peroxidase method (Ultravision Detection System Anti-Polyvalent, HRP by Lab Vision Corporation, and Liquid DAB Substrate Chromogen System by DAKO), to visualize antibody binding. Protein expression was then quantified by counting the percentage of positive cells per HPF in 10 randomly chosen HPFs.

### 2.3. *E. tirucalli* Treatment and Cell Proliferation

*E. tirucalli* plant extracts were prepared as described by Ito et al. [30]. Briefly, *E. tirucalli* extracts were obtained using 200 mL ether under reflux for 72 hours. The ethereal solution was then evaporated down and the resultant oily extracts were then dissolved in methanol and served as the test substance for EBV antigen activation. Cells were treated with different concentration of *E. tirucalli* (0.1, 0.5, 1, and 10 *μ*g/mL), resolved in methanol. As a normal control, untreated cells were cultured with the same amount of methanol. For the proliferation assay, cells were counted each day for 4 days. Statistical significance was assessed by the analysis of variance (ANOVA) test. For detection of EBV-specific responses, Ramos cells, an EBV-negative Burkitt lymphoma-derived cell line, were used as negative control and were cultured as previously described.

### 2.4. Cell Death Analysis

 Cell death was evaluated by several approaches. Cell viability was checked by Trypan Blue exclusion test. Cell cycle analysis was performed by flow cytometry on a FACStar (BD Bioscience, CA). Forward Scatter (FSC) and Side Scatter (SSC) signals were recorded in linear mode. Dead cells and debris were gated out using scatter properties of the cells and additionally using propidium iodide (PI) at a concentration of 1 *μ*g/mL. Data was analyzed using CellQuest software (BD Bioscience, CA). Apoptosis was detected by DNA laddering on a 1% agarose gel. Caspase staining for caspase 3 and 8 was detected by immunocytochemistry, as previously described.

### 2.5. Fluorescence *In Situ* Hybridization (FISH)

 Briefly, *MYC* and *BCL2* rearrangements were sought using the *MYC* FISH DNA Probe-Split Signal using standard procedures (*BCL2*, IgH, IgL, *BCL6*, break-apart probes, and *MYC* dual color probe). Briefly, smeared cells were air-dried, immersed in a jar filled with pretreatment solution, and warmed at 98°C for 10 min by means of a Whirlpool JT 356 microwave. Subsequently, the slides were cooled for 15 min at RT. After two passages of 3 min each in Wash Buffer, excess buffer was tapped off and the slides digested with cold Pepsin for 20 min in a Dako Cytomation Hybridizer (Dako, Denmark). The slides were then washed twice in Wash Buffer for 3 min, dehydrated using increasing graded ethanol series, air-dried, and finally 10 *μ*L of probe mix were applied to each tissue section. The slides, covered with coverslip and sealed with rubber cement, were then incubated in the DakoCytomation Hybridizer (Dako, Denmark) according to the manufacturer's recommendations. The next day, slides were treated with stringency buffer at 65°C for 2 min then placed twice in Wash Buffer for 3 min, dehydrated using increasing graded ethanol series, air-dried, and counterstained applying 15 *μ*L of Fluorescence Mounting Medium. Hybridization signals were visualized using a Leica microscope equipped with a triple-band filter for detecting green fluorescent protein (GFP)/spectrum green, Texas red/spectrum orange, and DAPI/spectrum blue. Images were captured and archived using Leica FW4000 software. One hundred nonoverlapping interphase nuclei were scored for each tumor specimen. In normal nuclei, two yellow fusion signals (2F) are detected, whereas in nuclei with translocations, a yellow (or red-green juxtaposed) signal is obtained from one red and one green segregated signal (1F1R1G). The results were further confirmed by additional FISH analysis using split-signal probes for IgH and IgL loci as well as an LSI *IGH*/*MYC* CEP 8 Tri-color dual-fusion probe (Vysis, Abbott Molecular IL, USA) specific for the detection of the translocation t[8; 14]. All reagents, instruments, and split-signal probes were kindly provided by DakoCytomation (Glostrup, Denmark). To specifically detect chromosome 8, the centromeric probe Zyto Light SPEC CMYC/CEN8 Dual Color Probe (ZytoVision, Germany) has been used. To establish the *MYC*:chromosome 8 ratio, 100 nuclei were randomly chosen and signals for *MYC* and chromosome 8 were counted.

### 2.6. qRT-PCR

Real-time PCR for *MYC* and *BCL2* was performed using FluoCycle SYBR green (Euroclone, Celbio, Italy) according to the manufacturer's instructions and *HPRT* as an internal control.

Primer sequences for *MYC* amplified a region of 129 bp: LEFT: AGCGACTCTGAGGAGGAAC; RIGHT: TGTGAGGAGGTTTGCTGTG. Primer sequences for *BCL2* amplified a region of 258 bp: LEFT: 5′-TTGCCACGGTGGTGGAGGA-3′; RIGHT: 5′-ACAGCCAGGAGAAATCAAACAG-3′. Primer sequences for *HPRT* amplified a region of 191 bp: LEFT: AGCCAGACTTTGTTGGATTTG; RIGHT: TTTACTGGCGATGTCAATAAG. Differences in gene expression were calculated using the ΔΔCt method [31].

### 2.7. Indirect Immunofluorescence

 For c-MYC detection, cells were smeared on positively charged slides after *E. tirucalli* treatment (Day 5) and fixed in 4% paraformaldehyde in PBS for 10 minutes at room temperature. Permeabilization was achieved by washing cells in PBS, 0.2% Triton X-100, and 1% BSA. Saturation was performed for 1 hour at room temperature in goat serum (Zymed laboratories, Milan, Italy). All of the antibodies were diluted in goat serum. Primary antibody incubation was carried out at room temperature for 1 hour, using anti-c-MYC (9E10 sc-40: Santa Cruz Biotechnology, Santa Cruz, CA) 1 : 50. Secondary goat anti-mouse antibody, conjugated with Alexafluor568 (Molecular Probes, Invitrogen, Milan, Italy), was diluted 1 : 100 in goat serum and incubated at room temperature for 45 minutes. The slides were examined on an Axiovert 200 microscope (Carl Zeiss, Germany) and processed with Zeiss software (Carl Zeiss, Germany). Nuclei were counterstained by DAPI.

## 3. Results

### 3.1. *E. tirucalli* Treatment Affects Cell Proliferation

**Figure 1:**
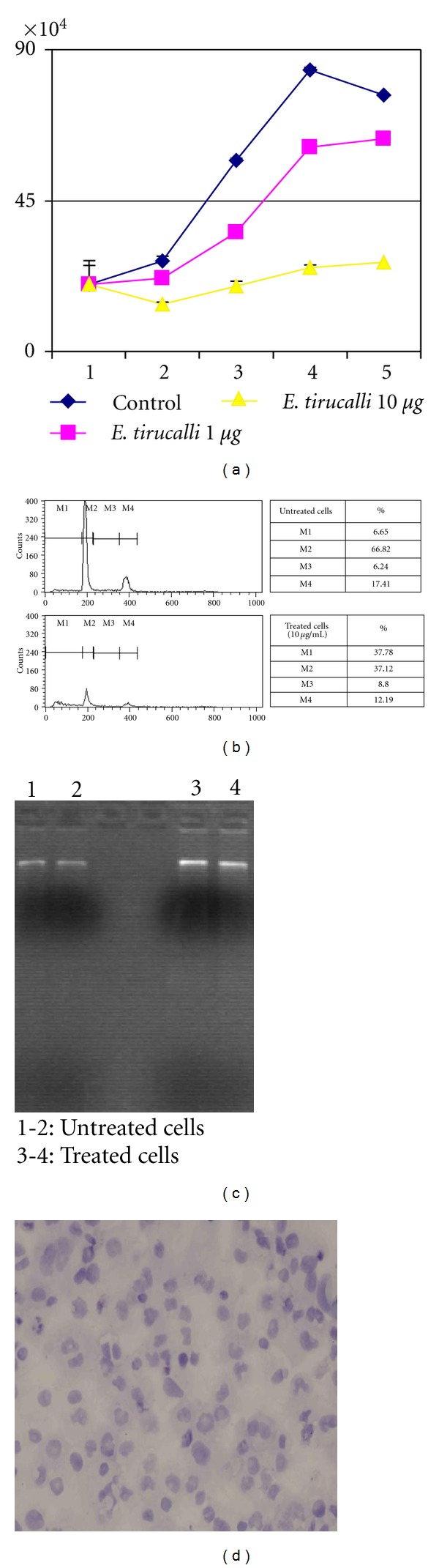
(a) Cord-blood-derived cells were treated with different concentration of *E. tirucalli* for five days, and proliferation has been monitored. Methanol-treated cells, with no *E. tirucalli* extract, have been used as a control. A dose-dependent decrease of proliferation rate is observed in *E. tirucalli*-treated cells (*P* < 0.05). The graph is representative of three different experiments. Error bars represent standard deviation between duplicates. (b) Cell cycle analysis by FACS on untreated (upper part) and *E. tirucalli*-treated cells (lower part). Tables indicate the percentage of cells in each cell cycle stages, where M1 indicates total number of dead cells (apoptotic and necrotic cells), M2 indicates G0/G1, M3 cells in S phase, and M4 the G2/M phase. Treated cells show a higher number of the M1 fraction. (c) Electrophoresis on agarose gel of untreated (lanes 1-2) and *E. tirucalli*-treated cells (lanes 3-4). No DNA laddering indicative of cell death by apoptosis is visible. (d) ICC for caspase 3. No caspase activation is detected following *E. tirucalli* treatment.

We treated LCLs and the cord-blood-derived cell line with increasing concentrations of *E. tirucalli*, as reported in Section 2, using methanol-treated cells as a control. The effects of the treatment on cell proliferation and cell death were monitored. A dose-dependent reduction in cell proliferation was observed after *E. tirucalli* treatment (Figure 1(a)), accompanied by high rates of cell deaths (Figure 1(b)).

**Figure 2:**
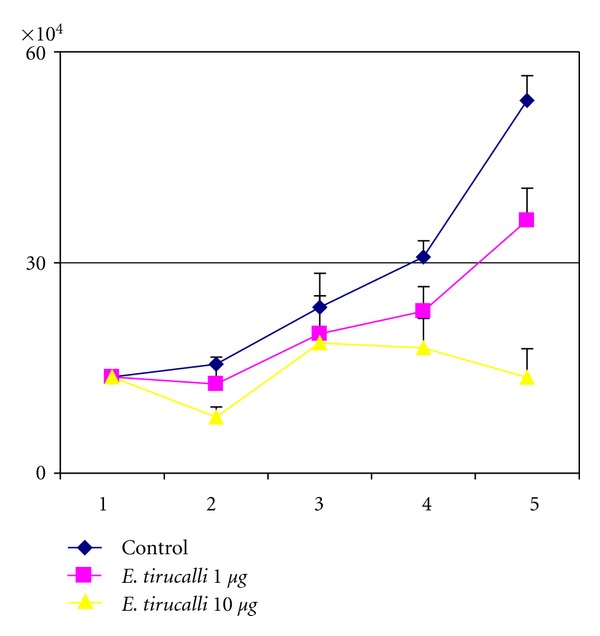
To rule out the possibility that the decrease of cell proliferation and the concomitant increase in cell death observed in *E. tirucalli*-treated cells was due to induction of the lytic pathway of EBV, an EBV-negative cell line (Ramos) was cultured in the same experimental conditions as cord blood derived cells. *E. tirucalli* treatment had the same effects on cell proliferation and cell death, independently of EBV status. Treated cells showed a decreased cell proliferation (*P* < 0.05). The graph is representative of three different experiments. Error bars represent standard deviation between duplicates.

Cell death seemed to be due to necrosis rather than to apoptosis, as no DNA laddering nor caspase activation was observed following *E. tirucalli* treatment (Figures 1(c) and 1(d)). To assess whether cell death was due to the toxicity effects of the plant extracts or to reactivation of EBV, we treated an EBV-negative cell line using the same experimental conditions, and we observed a similar reduction in cell proliferation accompanied by an increase in cell death suggesting that cell death was due to plant toxicity (Figure 2).

### 3.2. *E. tirucalli* Modulates the Expression of EBV-Antigens

**Figure 3:**
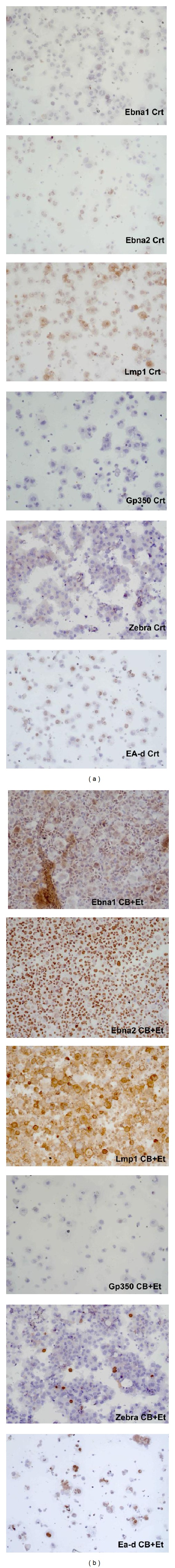
Immunocytochemistry of untreated (a) and *E. tirucalli*-treated cells (b). The expression of EBV-coded products was monitored.

There are three different latency programs of EBV, characterized by the differential expression of its coded genes [32]. In additions, the expression of some EBV-genes, such as Zebra, indicates the shift from the latent to the lytic phase of the virus [33]. Reactivation of EBV as a consequence of TPA-analogous treatment had been reported by literature [24]. We, therefore, treated cells with different concentrations of *E. tirucalli* and monitored the expression of EBV-coded genes by ICC. After *E. tirucalli* treatment, we observed the expression of Zebra, which was not expressed by untreated cells. In addition, enhanced expression of the EBV early antigen (EA), LMP1 and EBNA2 expression was also observed, whereas no significant variation of the EBV early antigen gp350 was detected following *E. tirucalli* treatment. Higher concentrations of plant extracts (10 *μ*g/mL) resulted in a more marked EBV protein expression. Comparable results were obtained in LCL and cord-blood cell lines.  Table 2 summarizes immunocytochemical results obtained in treated versus untreated cells.  Figure 3 shows ICC results.

### 3.3. *E. tirucalli* Induces Chromosome 8 Polysomy

**Figure 4:**
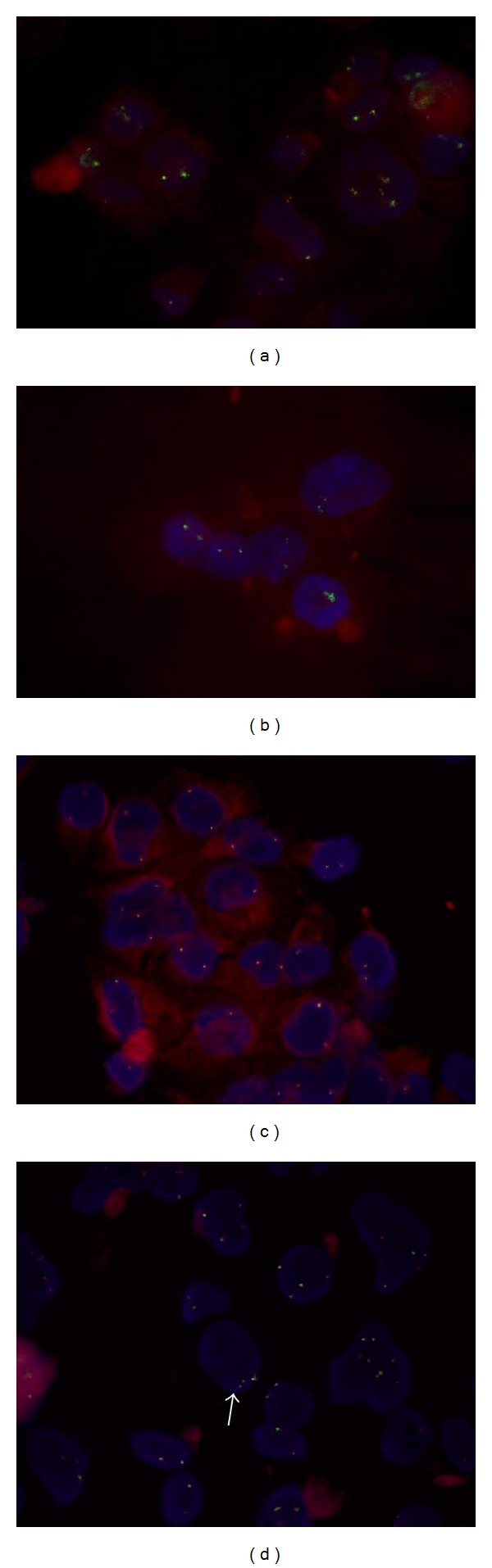
FISH analysis for *BCL2* (a, b) and *MYC* (c, d) for untreated (a, c) and *E. tirucalli*-treated cells (b, d). No balanced translocations have been detected, though multiple signals for *MYC* have been identified. Arrows indicate cells with multiple *MYC* signals.

*E. tirucalli* has been shown to induce genetic alterations, particularly those involving the oncogene *MYC *[27]. To detect the onset of specific chromosomal translocations after *E. tirucalli* treatment, we performed FISH analysis to identify the most frequently described chromosomal translocations occurring in aggressive B-cell lymphomas, using probes designed to detect *MYC*, *BCL-2, BCL6,* and their respective Ig partners. Using this approach, no balanced translocations involving *BCL2, BCL6, *IgH, and IgL were detected.  Figure 4 summarizes FISH results for *BCL2* and *MYC*.

**Figure 5:**
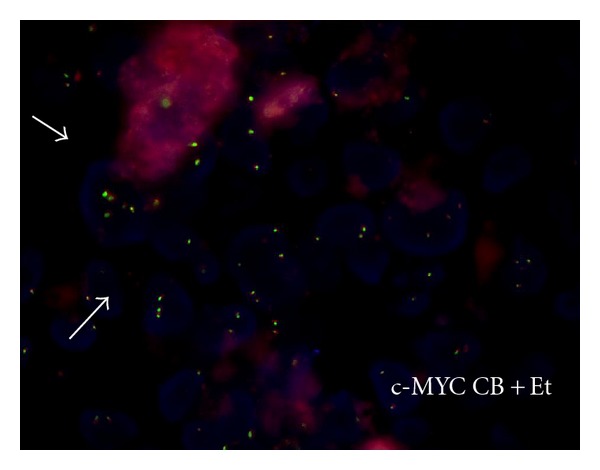
To assess whether multiple *MYC* signals relied on *MYC* gene amplification or on chromosomal 8 polyploidy, a centromeric probe for chromosome 8 was used. Our results indicated the presence of multiple signals for chromosome 8 in cells treated with *E. tirucalli*, consistent with a polysomy of chromosome 8. Arrows indicate cells with multiple chromosome 8 and *MYC* signals.

 Multiple signals for *MYC* were detected in about 17% of cells, ranging between 3 and more copies, in contrast to the normal 2 copies, though no specific chromosomal translocation could be identified. To assess whether these signals were dependent on gene amplification or to a chromosome 8 polysomy, we used a centromeric probe to detect both *MYC* and centromere signals. Our results indicated a chromosome 8 polysomy, as more than 2 signals for the centromeres were detected, together with multiple signals for *MYC* (Figure 5).  Table 3 summarizes FISH results. 

### 3.4. c-MYC Expression Is Upregulated following *E. tirucalli* Treatment

**Figure 6:**
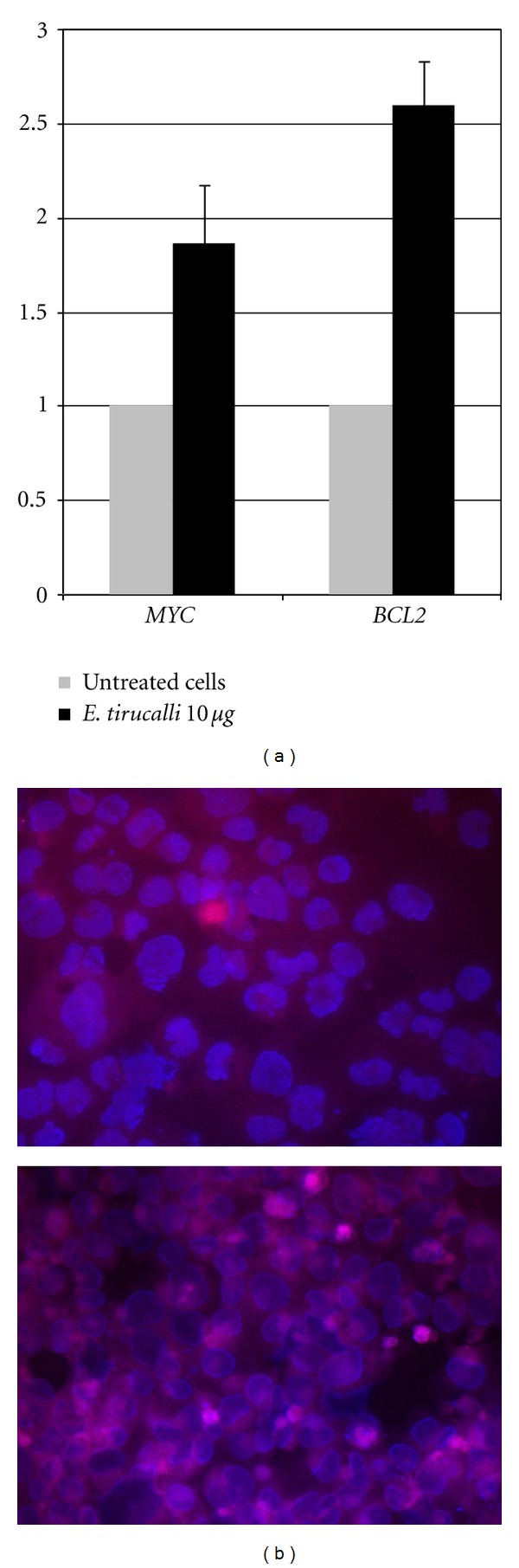
(a) qRT-PCR for *BCL2* and *MYC* in untreated and *E. tirucalli*-treated cells. A marked up-regulation of both genes is observed following treatment. The graph is representative of three different qRT-PCR experiments. Error bars represent standard deviation between duplicates. (b) Immunofluorescence of untreated (upper panel) or *E. tirucalli*-treated cells (lower panel). c-MYC expression increases following treatment. Magnification 40x.

The presence of multiple copies of *MYC* is compatible with an overexpression of the c-MYC protein, as occurs in Burkitt lymphoma. Therefore, we checked the expression level of c-MYC by qRT-PRC and immunofluorescence. As a consequence of chromosome 8 polysomy, upregulation of c-MYC was observed, as expected, at both levels (Figures 6(a) and 6(b)).

### 3.5. BCL2 Is Overexpressed following *E. tirucalli* Treatment

**Figure 7:**
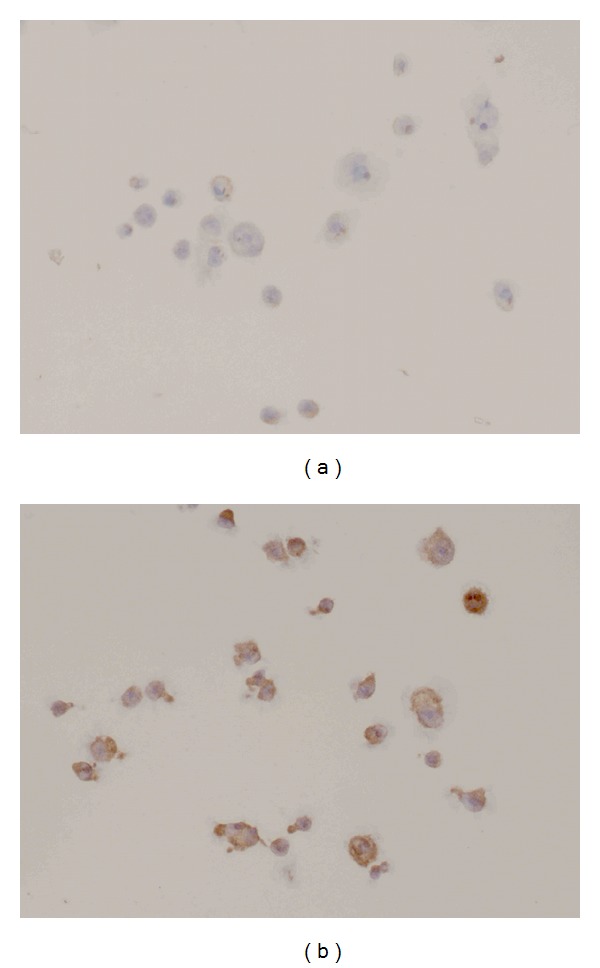
ICC for *BCL2 *in untreated (a) and *E. tirucalli*-treated cells (b). Magnification 40x.

*BCL2* deregulation is often observed in lymphomas, as in the case of follicular lymphoma. Though FISH revealed the absence of any chromosomal translocations involving *BCL2*, which maps on chromosome 18, we detected up-regulation of its expression level in treated cells, both at the mRNA (Figure 6(a)) and protein levels (Figure 7), which may be compatible with the acquirement of an anti-apoptotic capability by treated cells.

## 4. Discussion

 A potential transforming capability by *E. tirucalli* extracts has been suggested by a single publication in the last twenty years [27]. No further studies have been performed since then to highlight through which molecular mechanisms it occurred. In this study, we report the results on cell proliferation and cell death, expression of EBV-antigens, and induction of chromosomal abnormalities in LCL and cord-blood-derived cell lines after treatment with *E. tirucalli* plant extracts. Results have been almost completely matching between the two cell lines, though the freshly established cord-blood-derived cell line is more likely to represent the *in vivo* situation in respect with an LCL, as a prolonged *in vitro* culture in the latter could determine the accumulation of preexisting genetic abnormalities.

*E. tirucalli* treatment determined a reduction of cell proliferation and a concomitant increase of cell death. This result is in contrast to what reported by literature for PBMCs treated with *E. tirucalli* [34], which seems to result in an increased proliferation rate following treatment. Quite interestingly, we were not able to reproduce these results neither in LCLs or cord-blood-derived cells. One possible explanation could be that the increase of cell proliferation previously reported [34] has been observed mostly on the CD3^+^ subpopulation within PBMC, which represents lymphoid T-cells, whereas our results are referred to EBV-infected B-cells.

 Cell death was quite high in all the treated cells, being highest with the highest concentrations and seemed to depend on necrosis due to the plant toxicity, rather than to apoptosis, as no DNA laddering nor caspase activation was detected in cells treated with *E. tirucalli* (Figures 1(c) and 1(d)). The possibility that cell death was due to activation of the lytic cycle of EBV, as possibly suggested by Zebra expression, was ruled out by treating an EBV-negative cell line using the same experimental conditions. Cell death levels in this cell line were comparable to those observed in EBV-positive cells, thus suggesting that cell death was due to plant toxicity.

 On the contrary, treatment with *E. tirucalli* extracts modulated the expression of EBV-antigens. In particular, the expression of early antigens and a marked upregulation of LMP1 were observed after treatment. LMP1 expression may be relevant for NF-*κ*B activation [35] and apoptosis [36]. A similar pattern of expression of EBV antigens expression was observed in LCL and cord blood cells, with the exception of gp350, which was not expressed in cord-blood after treatment, whereas a weak expression was observed in LCL.

Although it has been previously reported that cells treated with *E. tirucalli* and cultured for one year accumulated genetic abnormalities, here we report for the first time that as less as a five-day treatment of *E. tirucalli* was able to determine genomic abnormalities both in LCL and CB. In particular, in both cases, polysomies were observed, being more evident the higher the concentration of *E. tirucalli* was. The possibility that the observed polysomies observed in LCL could be due to the accumulation of several genetic alterations, which may happen to cells cultured for many years, was ruled out by the observation that polysomies were induced by *E. tirucalli* treatment also in newly established CB cells, with a normal caryotype. In particular, polysomy of chromosome 8 was detected by FISH through specific centromeric probes, resulting in an increased number of copies of the *MYC* oncogene, as detected by FISH. Though Aya et al. reported the occurrence of specific chromosomal translocation in about 10% of treated cells after one year of culture, we did not detect any specific translocations. One possible explanation could be that our analyses have been performed after only five days of treatment, which were enough to induce polysomies, but may be not sufficient to let the chromosomal translocation occur. It is presumable that keeping these already genetically altered cells growing for a longer period of time might determine the onset and the accumulation of further genetic alterations, as translocations. In any case, the increased number of *MYC* copies could mimic the effects of the *MYC* activation due to the translocation, as observed in most BL. It is worth noting, nevertheless, that BL cases lacking *MYC* translocation do exist, in which the *MYC *expression level is increased due to different mechanisms [4, 5, 37]. Of interest, polysomies were detected only for chromosome 8, suggesting a predilection for a specific genetic locus alteration after *E. tirucalli* treatment.

 Interestingly, EBV reactivation may be crucial as its proteins may induce the expression of cellular genes. In particular, EBNA2 may induce *MYC* expression [38], whereas LMP1 may induce *BCL2* expression [39]. This may be of help to explain *BCL2* hyperexpression following *E. tirucalli* treatment, as no chromosomal translocations, neither genetic alterations involving *BCL2* have been detected. It is reasonable to hypothesize *BCL2* upregulation may be due to LMP1, whose expression is induced by *E. tirucalli*. In addition, EBNA2 overexpression, which is itself induced by *E. tirucalli,* may also contribute to hyperexpression of *MYC*. The observation of multiple signals for chromosome 8 indicates an additional mechanism explaining *MYC* upregulation, which can synergistically act with EBNA2-induced *MYC* expression, in determining higher expression levels of c-MYC. The overexpression of c-MYC should lead to both an increase of cell proliferation and cell death, as *MYC* has proliferative and proapoptotic effects, thus keeping balanced cell number. *E. tirucalli*-treated cells showed a marked upregulation of the antiapoptotic gene *BCL2*, though no genetic alterations for this gene had been detected by FISH. The upregulation of *BCL2* may counteract the proapoptotic effect due to c-MYC overexpression and could give the treated cells a growth advantage, which may contribute to malignant transformation.

 Collectively, our preliminary data suggest that *E. tirucalli* may cooperate in inducing malignant transformation, due to its modulation of the expression of the latency genes of EBV, and the upregulation of two key factors as *BCL-2 *and *MYC.* In particular, the overexpression of c-MYC seems to rely on the induction of polysomies after treatment, rather than chromosomal translocations.

 These observations suggest that *Euphorbia tirucalli* extracts and, possibly, extracts of other plants, which are known to have similar EBV-activating properties, could act as cofactors for lymphomagenesis in endemic African Burkitt's lymphoma.

## Figures and Tables

**Table 1:** List of antibodies and their respective concentrations.

Primary antibody	Dilution	Company
EBNA-1	1 : 50	Novus Biologicals
EBNA-2	1 : 50	Dako
ZEBRA	1 : 50	Santacruz
LMP1	1 : 50	Dako
EA	1 : 50	Santacruz
Gp350	1 : 100	Santacruz
BCL6	1 : 30	Dako
BCL2	1 : 150	Dako
CD20	1 : 150	NeoMarkers
CD27	1 : 50	NeoMarkers
IgM	1 : 10000	Dako
CD30	1 : 50	NeoMarkers
CD10	1 : 20	NeoMarkers
CD79	1 : 50	NeoMarkers
IgD	1 : 50	NeoMarkers
Irf-4	1 : 50	Dako
CD138	1 : 100	Dako
Caspase 3	1 : 50	Abcam

**Table 2:** Immunocytochemistry (ICC) of EBV-encoded proteins in cells treated with *E. tirucalli *at the concentration of 10 *μ*g/mL versus untreated cells, expressed as percentage of positive cells out of total cells. Comparable results were obtained in LCL and cord blood-derived cells.

	Control (%)	*E. tirucalli* 10 *μ*g/mL (%)
Zebra	0	6
Ea-d	40	50
LMP1	50	90
Gp350	0	0
EBNA1	5	30
EBNA2	10	80

**Table 3:** FISH analysis on *E. tirucalli* treated versus untreated cells.

	Control	*E. tirucalli* 10 *μ*g/mL
BCL2	No translocation	No translocation
BCL6	No translocation	No translocation
IgH (chromosome 14)	No translocation	No translocation
IgL (chromosome 22)	No translocation	No translocation
c-MYC (chromosome 8)	No translocation	Polysomies, no translocation

## References

[1] Leoncini L, Raphael M, Stein H (2008). *WHO Classification of Tumours of Hematopoietic and Lymphoid Tissues*.

[2] Burkitt D (1958). A sarcoma involving the jaws in African children. *British Journal of Surgery*.

[3] van den Bosch CA (2004). Is endemic Burkitt’s lymphoma an alliance between three infections and a tumour promoter?. *Lancet Oncology*.

[4] Leucci E, Cocco M, Onnis A (2008). *MYC* translocation-negative classical Burkitt lymphoma cases: an alternative pathogenetic mechanism involving miRNA deregulation. *Journal of Pathology*.

[5] Leucci E, Onnis A, Cocco M (2010). B-cell differentiation in EBV-positive Burkitt lymphoma is impaired at posttranscriptional level by miRNA-altered expression. *International Journal of Cancer*.

[6] Epstein MA, Robinson ES (2005). The origins of EBV research discovery and characterization of the virus. *Epstein-Barr Virus*.

[7] Lenoir GM, Preud’homme JL, Bernheim A, Berger R (1982). Correlation between immunoglobulin light chain expression and variant translocation in Burkitt’s lymphoma. *Nature*.

[8] Niller HH, Salamon D, Ilg K (2003). The in vivo binding site for oncoprotein c-*MYC* in the promoter for Epstein-Barr virus (EBV) encoding RNA (EBER) 1 suggests a specific role for EBV in lymphomagenesis. *Medical Science Monitor*.

[9] Moormann AM, Chelimo K, Sumba OP (2005). Exposure to holoendemic malaria results in elevated Epstein-Barr virus loads in children. *Journal of Infectious Diseases*.

[10] Magrath I, Jain V, Bhatia K (1992). Epstein-Barr virus and Burkitt’s lymphoma. *Seminars in Cancer Biology*.

[11] de Thé G, Lenoir G, O’Conor G, Olweny CLM (1985). Epstein-Barr virus and Burkitt’s lymphoma worldwide: the casual relationship revisited. *A Human Cancer Model, Burkitt’s Lymphoma*.

[12] de Thé G (1997). Epstein_Barr virus and associated diseases. Course of medical virology, institut pasteur, 1995-6. *Annales de Médecine Interne (Paris)*.

[13] de Thé G, Geser A, Day NE (1978). Epidemiological evidence for causal relationship between Epstein-Barr virus and Burkitt’s lymphoma from Ugandan prospective study. *Nature*.

[14] Levine PH, Fraumeni JF, Reisher JI, Waggoner DE (1974). Antibodies to Epstein Barr virus associated antigens in relatives of cancer patients. *Journal of the National Cancer Institute*.

[15] Hildesheim A, West S, DeVeyra E (1992). Herbal medicine use, Epstein-Barr virus, and risk of nasopharyngeal carcinoma. *Cancer Research*.

[16] Ito Y, Epstein MA, Achong BG (1985). Vegetable Activators of the viral genome and the Causations of Burkitt’s lymphoma and Nasopharyngeal carcinoma. *The Epstein-Barr Virus: Recent Advances*.

[17] van den Bosch C, Griffin BE, Kazembe P, Dziweni C, Kadzamira L (1993). Are plant factors a missing link in the evolution of endemic Burkitt’s Lymphoma?. *British Journal of Cancer*.

[18] Osato T, Imai S, Koizumi S (1987). African Burkitt’s lymphoma and an Epstein-Barr virus-enhancing plant Euphorbia triucalli. *The Lancet*.

[19] Osato T (1998). Epstein-Barr virus infection and oncogenesis. *Epstein-Barr Virus and Human Cancer. Gann Monograph on Cancer Research no. 45*.

[20] Furstenberger G, Hecker E (1985). On the active principles of the spurge family (Euphorbiaceae). XI. [1] The skin irritant and tumor promoting diterpene esters of *Euphorbia tirucalli* L. originating from South Africa. *Zeitschrift fur Naturforschung C*.

[21] Mizuno F, Koizumi S, Osato T (1983). Chinese and African Euphorbiaceae plant extracts: markedly enhancing effect on Epstein-Barr virus-induced transformation. *Cancer Letters*.

[22] MacNeil A, Sumba OP, Lutzke ML, Moormann A, Rochford R (2003). Activation of the Epstein-Barr virus lytic cycle by the latex of the plant *Euphorbia tirucalli*. *British Journal of Cancer*.

[23] Imai S, Sugiura M, Mizuno F (1994). African Burkitt’s lymphoma: a plant, *Euphorbia tirucalli* reduces Epstein-Barr virus-specific cellular immunity. *Anticancer Research*.

[24] Kanamori M, Tajima M, Satoh Y (2000). Differential effect of TPA on cell growth and Epstein-Barr virus reactivation in epithelial cell lines derived from gastric tissues and B cell line Raji. *Virus Genes*.

[25] Neuwinger HD (1996). Euphorbia tirucalli. *African Ethnobotany: Poisons and Drugs. Chemistry, Pharmacology, Toxicology*.

[26] Davies AH, Grand RJA, Evans FJ, Rickinson AB (1991). Induction of Epstein-Barr virus lytic cycle by tumor-promoting and non-tumor-promoting phorbol esters requires active protein kinase C. *Journal of Virology*.

[27] Aya T, Kinoshita T, Imai S (1991). Chromosome translocation and c-*MYC* activation by Epstein-Barr virus and *Euphorbia tirucalli* in B lymphocytes. *The Lancet*.

[28] Steel CM, Morten JE, Foster E (1985). The cytogenetics of human B lymphoid malignancy: studies in Burkitt’s lymphoma and Epstein-Barr virus-transformed lymphoblastoid cell lines. *IARC Scientific Publications*.

[29] Pelloquin F, Lamelin JP, Lenoir GM (1986). Human B lymphocytes immortalization by Epstein-Barr virus in the presence of cyclosporin A. *In Vitro Cellular and Developmental Biology*.

[30] Ito Y, Kawanishi M, Harayama T, Takabayashi S (1981). Combined effect of the extracts from Croton tiglium, Euphorbia lathyris or *Euphorbia tirucalli* and n-butyrate on Epstein-Barr virus expression in human lymphoblastoid P3HR-1 and Raji cells. *Cancer Letters*.

[31] Livak KJ, Schmittgen TD (2001). Analysis of relative gene expression data using real-time quantitative PCR and the 2-ΔΔCT method. *Methods*.

[32] Thorley-Lawson DA, Gross A (2004). Persistence of the Epstein-Barr Virus and the Origins of Associated Lymphomas. *New England Journal of Medicine*.

[33] Hsu CH, Hergenhahn M, Chuang SE (2002). Induction of Epstein-Barr virus (EBV) reactivation in Raji cells by doxorubicin and cisplatin. *Anticancer Research*.

[34] Llanes-Coronel DS, Gámez-Díaz LY, Suarez-Quintero LP (2011). New promising Euphorbiaceae extracts with activity in human lymphocytes from primary cell cultures. *Immunopharmacology and Immunotoxicology*.

[35] Laherty CD, Hong Ming Hu, Opipari AW, Wang F, Dixit VM (1992). The Epstein-Barr virus LMP1 gene product induces A20 zinc finger protein expression by activating nuclear factor *κ*B. *Journal of Biological Chemistry*.

[36] de Leo A, Arena G, Stecca C, Raciti M, Mattia E (2011). Resveratrol inhibits proliferation and survival of Epstein Barr virus-infected Burkitt's lymphoma cells depending on viral latency program. *Molecular Cancer Research*.

[37] Anna O, Giulia DF, Cristiana B, Federica M, Emily R, Lorenzo L MicroRNA expression profile identifies a different signature between *MYC* translocation-positive and negative Burkitt Lymphoma cases. New insights into the miRNA regulation relied on *MYC* pseudogene expression.

[38] Kaiser C, Laux G, Eick D, Jochner N, Bornkamm GW, Kempkes B (1999). The proto-oncogene c-*MYC* is a direct target gene of Epstein-Barr virus nuclear antigen 2. *Journal of Virology*.

[39] Henderson S, Rowe M, Gregory C (1991). Induction of bcl-2 expression by Epstein-Barr virus latent membrane protein 1 protects infected B cells from programmed cell death. *Cell*.

